# Uropathogenic *Escherichia coli* Biofilm-Forming Capabilities are not Predictable from Clinical Details or from Colonial Morphology

**DOI:** 10.3390/diseases8020011

**Published:** 2020-04-30

**Authors:** Shane Whelan, Mary Claire O’Grady, Dan Corcoran, Karen Finn, Brigid Lucey

**Affiliations:** 1 Department of Biological Sciences, Cork Institute of Technology, Bishopstown, T12P928 Cork, Ireland; Swhelan1@mycit.ie; 2 Department of Clinical Microbiology, Cork University Hospital, Wilton, T12 DC4A Cork, Ireland; m.ogrady@mycit.ie (M.C.O.); Dan.Corcoran@hse.ie (D.C.); 3 Department of Biological Sciences, Galway-Mayo Institute of Technology, Old Dublin Road, H91 DCH9 Galway, Ireland; Karen.Finn@gmit.ie

**Keywords:** uropathogen, *Escherichia coli*, biofilm formation, colony morphology, urinary tract infection

## Abstract

Antibiotic resistance is increasing to an extent where efficacy is not guaranteed when treating infection. Biofilm formation has been shown to complicate treatment, whereby the formation of biofilm is associated with higher minimum inhibitory concentration values of antibiotic. The objective of the current paper was to determine whether biofilm formation is variable among uropathogenic *Escherichia coli* isolates and whether formation is associated with recurrent urinary tract infection (UTI), and whether it can be predicted by phenotypic appearance on culture medium A total of 62 *E. coli* isolates that were reported as the causative agent of UTI were studied (33 from patients denoted as having recurrent UTI and 29 from patients not specified as having recurrent UTI). The biofilm forming capability was determined using a standard microtitre plate method, using *E. coli* ATCC 25922 as the positive control. The majority of isolates (93.6%) were found to be biofilm formers, whereby 81% were denoted as strong or very strong producers of biofilm when compared to the positive control. Through the use of a Wilcox test, the difference in biofilm forming propensity between the two patient populations was found to not be statistically significant (*p* = 0.5). Furthermore, it was noted that colony morphology was not a reliable predictor of biofilm-forming propensity. The findings of this study indicate that biofilm formation is very common among uropathogens, and they suggest that the biofilm-forming capability might be considered when treating UTI. Clinical details indicating a recurrent infection were not predictors of biofilm formation.

## 1. Introduction

Urinary tract infections (UTI) are among the most common infections affecting both men and, particularly, women, 50–60% of whom will at some time become infected with uropathogenic bacteria [1]. Enterobacteriaceae, including *Escherichia coli*, are the most common uropathogens, accounting for 80% of all reported infections [2]. While most cases of UTI can be successfully treated with oral antibiotics, recurrent infections are not uncommon, and several studies have pointed to biofilm formation and the associated antimicrobial resistance as a key factor leading to recurrence in UTIs. The majority of recurrent infections have previously been shown to be caused by biofilm positive strains [3], and it has been stated that the point of recurrence might be the bladder, as biofilm formation assists the organism in its colonisation, forming intracellular pod like biofilm structures within the bladder epithelial cells [4]. The treatment of biofilm associated UTI is further complicated due to the high levels of antibiotic resistance as compared to cells in a planktonic state [5].

Some antibiotics have been shown to be more effective than others when treating biofilm associated infections due to differences in mechanisms of action and ability to penetrate bladder urothelial cells to destroy inter-dwelling cell reservoirs. The cell wall synthesis inhibitors penicillin G, cefadroxil, and fosfomycin have been shown to inhibit biofilm development and eradicate pre-existing biofilm communities in uropathogenic *Escherichia coli* (UPEC), while the β-lactam antibiotic nafcillin was found to enhance biofilm formation [6]. A recent paper by our group on the subject of antimicrobial resistance rates among uropathogenic *E. coli* found that only one of six commonly-used oral antibiotics (nitrofurantoin) showed a resistance rate of <20% among all patient groups, which suggests that empirical treatment of UTI is likely to fail [7]. Therefore, it could useful to determine the biofilm forming propensity of UPEC prior to recommending treatment, so that the choice of antibiotic and regime time can be best directed to prevent the selection of resistant pathogens and reoccurrence of infection.

We sought to investigate the prevalence of biofilm formation among uropathogenic *E. coli* (UPEC) from patients with UTI whose clinical details suggested either recurrent UTI or non-recurrent infection, and to investigate whether colonial phenotypes might be a predictor of biofilm forming propensity, as inappropriate antibiotic treatment is associated with the development of antibiotic resistance, and the efficacy of antibiotics is significantly reduced when treating biofilm associated infections.

## 2. Materials and Methods

A total of 62 urines from patients with laboratory-confirmed UTI were provided by the Department of Clinical Microbiology, Cork University Hospital during 2019. Cork Research Ethics Committee granted ethical approval for this study (ECM4(q) 27/05/2019). The samples for the study were selected on the basis that they contained a pure culture of *E. coli.* These strains were identified to species level using Matrix Assisted Laser Desorption/Ionisation Time of Flight (MALDI-TOF; Microflex Biotyper, Bruker Daltronics, Hamburg, Germany) at Cork university hospital). 

Fresh overnight isolates of the test strains were identified while using MALDI-TOF. A colony of the test sample was spotted onto the mass spectra (MSP) (main spectra library) 96 target polished steel plate (Bruker Daltronics). Formic acid was added to the test colonies. The samples were then air-dried at room temperature and overlaid with 1 mL Bruker HCCA matrix solution (a-Cyano-4-hydroxycinnamic acid). Each test strain was analysed in duplicate. The results for each strain were matched to the Bruker database, and an algorithm score was assigned to each generated result. Based on the peak that was observed for each test strain, a logarithmic score was given, which ranged from 0 to 3.0. The interpretative guidelines for the scores generated were as follows: A score of 0 to 0.1699 indicated that a reliable identification was not possible. A score of 1.700 to 1.999 was indicative of a probable genus identification. A score of 2.0 to 2.299 meant a definite genus identification and probable species identification. A score of 2.300 to 3.0 meant a highly probable species identification. All were reliably identified as *E. coli.*

Twenty-nine of the urines were from patients with no clinical details indicating recurrent urinary tract infection (unspecified population), while the remaining 33 urines were from patients whose clinical details indicated a recurrent infection (RUTI population). All of the specimens were cultured using Cysteine Lactose Electrolyte Deficient (CLED) agar and isolates were photographed to record colonial morphology in each case. *E. coli* ATCC 25922, previously described as a strong biofilm former [8], was used as a positive control strain for biofilm formation.

Biofilm formation was determined using the microtitre plate method [9]. Fresh colonies of each test sample were prepared by plating on Luria-Bertani (LB) agar and incubating at 37 °C for 24 h. The suspensions were then prepared by adding colonies to sterile ringers’ solution to an optical density equal to 0.5 MacFarland standard; 1μL of suspension was added to three wells of a 96-well microtitre plate containing 200μL of sterile LB broth for each test organism and *E. coli* 25922. A set of three wells containing LB broth were left uninoculated to serve as both a sterility control and as a blank to account for the unspecified binding of media. Plates were then incubated for 24 h at 37 °C. Media and unattached cells were removed by turning the plate upside down and gently tapping it and wells were washed three times with 0.8% sterile saline solution. The attached cells were then heat fixed to the microtitre plate by placing them in a 50 °C oven for 1 h. The affixed cells were then stained by adding 220 μL of 5% crystal violet (*v*/*v*) and incubated at room temperature for 20 min. The excess crystal violet was then washed away with 0.8% saline. The remaining crystal violet was dissolved by adding 220 μL of 30% acetic acid to each well and then incubating at room temperature for 20 min. An automatic plate reader was then used to determine the absorbance at 590 nm. This process was repeated, so that each strain was tested in triplicate, a total of three times, beginning with a separate fresh overnight culture each time.

The absorbance of the uninoculated blank wells were subtracted from the absorbance of each strain and the average among replicates was calculated along with the Standard Deviation (SD) to determine test variability to calculate biofilm formation. The average biofilm formation found for *E. coli* ATCC 25922 was designated a score of 1 and each test strain was given a proportional score, so that a score of 2 would indicate biofilm formation twice that of the control and a score of 0.5 would indicate biofilm formation 50% of the control, and so on. Isolates which scored between 0.9 and 2.0 were noted as strong biofilm formers, with isolates forming more than 2.0 noted as very strong. Weak biofilm formers were determined to be strains that scored less than 0.9, but at least 0.4 and those that generated less than 0.4 were arbitrarily designated as +/− (indeterminate)biofilm-formers. The colony morphology of each isolate as it appeared on CLED agar was noted along with the biofilm formed to determine whether the biofilm forming propensity was predictable based on colony appearance (see Table 1).

## 3. Results

Strong or very strong biofilm formation, which was characterised by producing at least 90% of the biofilm formed by the positive control ATCC 25922, was observed in 81% of the 62 isolates studied. Among the RUTI population, 73% were found to be either strong or very strong biofilm formers, where the biomass produced was equal to or exceeded the control organism, while, among the unspecified population, 90% were found to be strong or very strong. A total of 93.6% of all test isolates were found to be positive for biofilm formation, with the remainder (four isolates) showing indeterminate or very weak biofilm formation. A moderate level of variability was seen in the biofilm formation of individual strains between repeated runs, although the averages remain an accurate depiction of their biofilm forming tendencies. Notable isolates that may be considered hyper producers of biofilm were found between both populations. CIT30, isolated from a patient where a recurrent infection had been noted produced 12.8 times the average biomass of the control organism, while CIT9, which was isolated from a patient where recurrence was not specified produced 7.7 times the average biomass of the control. Through the use of a Wilcox test the difference in biofilm formation between the two populations was compared and found to not be statistically significant (*p* = 0.5). 

The size and shape of the colonies that formed by each isolate were examined to determine whether there existed a correlation that could be used to accurately predict the biofilm forming tendencies of UPEC isolates prior to treatment in a clinical setting. A Kruskal–Wallis test was performed finding no significant correlation between colony size and biofilm formation (*p* = 0.1). Non-lactose fermentation, which was noted in four of the isolates (6.5%), was associated with three strong or very strong biofilm-formers and one indeterminate or very weak biofilm-forming isolate. Statistical analysis of these phenotypes in relation to biofilm formation could not assessed with only two strains of UPEC noted as being mucoid, and the majority of strains being lactose fermenting.

## 4. Discussion

Biofilms provide extrinsic resistance by blocking the penetration of antimicrobials to the cells within [10], and intrinsic resistance—where the cellular envelope that forms the target of many antimicrobials are altered within the biofilm to inhibit antimicrobial action [11]. This resistance is coupled with a slower growth rate, nullifying certain antimicrobials that require a fast-growing organism to be effective, the end result of which are communities that can withstand antimicrobial concentrations of at least 1 × 10^3^ times above the MIC noted for the planktonic state [12]. The determination of biofilm-forming capability among UPEC in the current study was sparked by recent research by this research group, in which current empirical treatment guidelines were shown to be undermined by having high levels of resistance among UPEC, to the extent that it was necessary to recommend laboratory-guided treatment of all patients with suspected UTI [7]. Therefore, it was worth investigating whether the phenotypic appearance of *E. coli* might possibly be linked to biofilm-forming propensity, which in turn might point towards persistence of infection. It has been previously noted that UPEC infections can persist within the bladder, even after the completion of antibiotic treatment and the association of biofilm-like communities to the urothelium wall, in part, aids this persistence [6]. 

Biofilm formation is one component of multiple discernible microbial factors that can make recurrence more likely, with the process itself being linked to as many as 110 genes in *E.coli* [13]. Notably, the yersiniabactin (*fyn*) gene and the aerobactin (*aer*) gene were found to be frequent among strains leading to recurrence [3]. Therefore, it might then be an important clinical consideration when deciding on a treatment plan, but the large number of genes previously suggested to be associated with biofilm production suggests that molecular-based biofilm assays are unlikely to be used in the diagnostic laboratory in the immediate future. The importance of biofilm estimation [10,11,12] is underlined by the finding of several extremely high biofilm formers among the population in Table 1, most notably CIT30 found in a patient with RUTI, which formed 12.8 times the biomass of the control strain. The high level of positive strains found among the population is not unusual in studies of this type, where typically 69–92% of UPEC have previously been reported as being positive for biofilm formation [14,15,16]. A significant statistical difference in biofilm formation was not found between the two populations in the current study. However, the chance of recurrence after initial infection is high, with one prospective study conducted in Finland finding 44% of female patients developing RUTI one month after initial infection [17]. This implies that many of the unspecified strains may recur. No link was found between mucoidy, colony size as it appears on CLED agar, or lactose fermentation due to the predominant uniformity of the isolates, showing the inutility of phenotypic predictors in this setting, unfortunately. One potential weakness of the study was that it was not possible to contact the requesting clinician to determine whether all of the relevant clinical details were filled in on the request form to ensure that, where RUTI was not indicated, there were no omissions and, ideally, the authors would also like to examine more isolates while concurrently examining for any differences associated with patient gender, given that UTI is more common among females.

## 5. Conclusions

This work indicates that phenotypic colonial appearance does not predict biofilm forming capability. The work also shows that the capacity to form significant amounts of biofilm (at least in vitro), while prevalent, is not universal among UTI-associated strains of *E. coli*, whether from patients with recurrent or non-recurrent UTI. 

## Figures and Tables

**Table 1:** Biofilm production expressed as a factor of the control organism *E. coli* ATCC 25922 with colony morphology description.

Unspecified				Recurrent			
Isolate	Score	Biofilm Designation	Colony Description	Isolate	Score	Biofilm Designation	Colony Description
CIT1	1.6	Strong	2L	CIT30	12.8	Very strong	2L
CIT2	1.9	Strong	2L	CIT31	2.5	Very strong	2L
CIT3	1.6	Strong	2L	CIT32	2.2	Very strong	1L
CIT4	2.2	Very strong	2L	CIT33	0.6	Weak	2L
CIT5	0.8	Weak	2L	CIT34	0.3	+/−	2L
CIT6	5.8	Very strong	3LM	CIT35	0.3	+/−	3N
CIT7	1.5	Strong	2L	CIT36	0.4	Weak	1L
CIT8	0.9	Strong	2L	CIT37	3.2	Very strong	2L
CIT9	7.7	Very strong	2L	CIT38	5.5	Very strong	3N
CIT10	1	Strong	2L	CIT39	0.7	Weak	3L
CIT11	1.5	Strong	2L	CIT40	2.3	Very strong	2L
CIT12	1.1	Strong	2L	CIT41	1.4	Strong	2L
CIT13	1.4	Strong	2L	CIT42	1.1	Strong	3L
CIT14	1.2	Strong	3LM	CIT43	0.4	Weak	2L
CIT15	2.4	Very strong	2L	CIT44	0.9	Strong	2L
CIT16	2.7	Very strong	2L	CIT45	1.1	Strong	2L
CIT17	1.6	Strong	2L	CIT46	1.1	Strong	2L
CIT18	2.3	Very strong	2L	CIT47	3.4	Very strong	2N
CIT19	1.4	Strong	2N	CIT48	1.0	Strong	2L
CIT20	1.5	Strong	2L	CIT49	1.5	Strong	3L
CIT21	1.4	Strong	2L	CIT50	0.6	Weak	2L
CIT22	1.6	Strong	2L	CIT51	2.1	Strong	2L
CIT23	0.1	+/−	1L	CIT52	1.6	Strong	2L
CIT24	0.9	Strong	3L	CIT53	1.5	Strong	2L
CIT25	0.3	+/−	3L	CIT54	0.8	Weak	2L
CIT26	1.1	Strong	3L	CIT55	0.9	Strong	2L
CIT27	1.2	Strong	2L	CIT56	1.1	Strong	2L
CIT28	0.9	Strong	2L	CIT57	0.8	Weak	1L
CIT29	0.9	Strong	2L	CIT58	1.2	Strong	2L
25922	1	Strong	2L	CIT59	2.3	Very strong	2L
				CIT60	1.1	Strong	2L
				CIT61	0.9	Strong	2L
				CIT62	0.9	Strong	2L

Colony description key: 1, 2, 3 = small (<2 mm), medium (2–3 mm), large sized colonies (>3 mm). *L = lactose fermenting N = Non-lactose fermenting M = mucoid variant.*
